# Description of the inflammatory bowel disease natural history in Tehran province, Iran: Mixed panel approaches 

**Published:** 2021

**Authors:** Meysam Olfatifar, Hamid Asadzadeh Aghdaei, Mohamad Amin Pourhoseingholi, Hedieh Balaii, Saeed Hashemi Nazari, Shabnam Shahrokh, Siamak Sabour, Maria Ivanchuk, Pavlo Ivanchuk, Soheila Khodakarim, Mohammad Reza Zali, Pejman Rohani, Gholamhossein Mehralian

**Affiliations:** 1 *Gastroenterology and Liver Diseases Research Center, Research Institute for Gastroenterology and Liver Diseases, Shahid Beheshti University of Medical Sciences, Tehran, Iran*; 2 *Basic and Molecular Epidemiology of Gastrointestinal Disorders Research Center, Research Institute for Gastroenterology and Liver Diseases, Shahid Beheshti University of Medical Sciences, Tehran, Iran*; 3 *Prevention of Cardiovascular Disease Research Centre, Department of Epidemiology, School of Public Health and Safety, Shahid Beheshti University of Medical Sciences, Tehran, Iran*; 4 *Department of Clinical Epidemiology, School of Public Health and Safety, Shahid Beheshti University of Medical Sciences, Tehran, Iran.*; 5 *Safety Promotions and Injury, Prevention Research Centre, Shahid Beheshti University of Medical Sciences, Tehran, Iran *; 6 *Biological Physics and Medical Informatics Department, Bukovinian State Medical University, Chernivtsi, Ukraine*; 7 *Internal Medicine, Physical Rehabilitation, Sports Medicine and Physical Training Department, Bukovinian State Medical University, Chernivtsi, Ukrain*; 8 *Department of Biostatistics, School of Medicine, Shiraz University of Medical sciences, Shiraz, Iran *; 9 * Pediatric Gastroenterology and Hepatology Research Center, Pediatrics Centre of Excellence, Pediatric's Medical Center, Tehran University of Medical Sciences, Tehran, Iran*; 10 *School of Pharmacy, Shahid Beheshti University of Medical Sciences, Tehran, Iran*

**Keywords:** Inflammatory bowel disease, Multi-state model, Panel data, Crohn's disease, Ulcerative colitis

## Abstract

**Aim:**

Description of the inflammatory bowel disease natural history in Tehran province.

**Background:**

Inflammatory bowel disease (IBD) is a non-homogeneous disorder with an unpredictable natural history that impairs a patient's quality of life over the course of their life. As a result, providing evidence for efficient patient management is critical.

**Methods:**

In this case series study, 198 IBD patients who were visited in our clinic at least three times routinely from Oct 2015 to May 2020 were included. Then, two panel-based approaches, the Multi-State Model (MSM) and random-effect ordered logistic, were used to deduce the clinical course of IBD, which included remission, mild, moderate to severe, and surgical states.

**Results:**

For ulcerative colitis (UC), women had a slightly poorer condition for remission but better for moderate to severe and a faster transition from moderate to severe to mild (HR=1.490, 95% CI: 1.02-2.16) compared to men. For Crohn's disease (CD), they had a better condition for remission but a slightly poorer condition for the severe state and higher transition from mild to moderate to severe (HR=1.221, 95% CI: 0.471-3.22) than men. Oral 5-ASA had better efficacy in people with remission and/or mild states but not for those with moderate to severe states, especially in CD (mild to moderate to serve, HR=1.526, 95% CI: 0.59-3.89). Immunosuppressive drugs were better for patients with lower disease severity, especially with UC (mild to remission, HR=1.258, 95% CI: 0.75-2.09).

**Conclusion:**

Panel approaches have the potential efficacy to tackle the unpredictable clinical course of IBD (UC/CD). Hence, we highly recommend that our findings be included into the Iranian routine clinical environment of IBD and/or that related studies be conducted in Iran and other regions to gain a better understanding of the natural history of IBD.

## Introduction

 For chronic conditions such as inflammatory bowel diseases (IBDs), the natural history of the disease can be modeled on a set of finite states to elucidate both the natural history of the disease and its evolution over time (1). There are several classification systems for IBD and its two distinct subtypes, Ulcerative Colitis (UC) and Crohn's disease (CD). Patients are generally classified into several distinct states, such as remission, mild, moderate, or severe, based on disease extension and/or severity (2). However, the clinical course of IBD (UC/CD) remains uncertain (3) and is affected by a variety of clinical, serological, endoscopic, and genetic factors (4). Factors such as age (5), gender (5), smoking, drugs, C-reactive protein (6), and erythrocyte sedimentation rate can be considered (7). Identifying these factors in the clinical environment is critical for inducing sustained remission and preventing disease progression (4). Although the impacts of these factors have been examined in recent studies, there is still no suitable tool (4) for quantifying their individual and sometimes interactive effects. One useful method for this is the multi-state model (MSM) (1).

MSM provides additional insight into the natural history of chronic diseases (8, 9), like IBD, where patients are typically visited periodically for follow-up. In such case, not only are the details between visits unavailable, but the disease onset is also unclear (10). This model can also provide a better picture of both the treatment effects (11) and the variation in disease course between patients by adjusting for a particular collection of covariates. In this way, MSM can take a personalized approach, at least for a class of patients (12), as IBD is a non-homogenous condition and differs substantially among patients (13). These models help the physician make the best decision about the type and timing of treatment, which can lead to patients experiencing a prolonged recovery time.

Herein, to elucidates the clinical course of IBD in Iranian patients, the current study applied a multi-state model (continuous-time Markov) and a logistic panel to address the unclear natural history of IBD and attempted to explore the role of some of the underlying factors (age and sex). 

## Methods

***Patient's information***

In this case series analysis we enrolled all IBD cases referred to the Institute of Gastroenterology and Liver Disease Research at the Shahid Beheshti University of Medical Sciences from October 2015 to May 2020. Patients were over 15 years of age and had a history of at least three visits. For each patient, data on age, weight, sex, date of visit, residence (urban/rural), type of disease, state of disease (as described in the natural history section), and patient medications was recorded. We considered the time unit of the study to be the month.

***Natural history***

**Figure 1:**
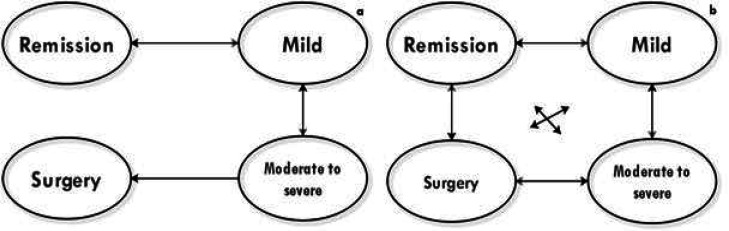
Inflammatory bowel disease natural history: an (ulcerative colitis) and b (Crohn's disease)

In our clinic, the Mayo classification system for UC (14) and the Crohn's Disease Activity Index (CDAI) (15, 16) for CD were used. Thus, this study employed the following model for UC and CD (Fig. 1). Remission (Mayo score 0-2 and CDAI<150), mild (Mayo score 3-5 and 150≤ CDAI≤220), and moderate to severe (Mayo score 6-12 and 220≤ CDAI≤600) were defined for UC and CD based on the Mayo and CDAI score systems, respectively. The state of surgery was also considered in the model but not death state, as no deaths in UC patients and only two deaths among CD cases were observed (In this situation, the model did not fit well) (Fig. 1).

***Drugs***

In general, three drug families were used in the management of IBD in our clinic as follows: 5-ASA or 5-aminosalisilicacid drugs (17): Asacol, Pentasa, Sulfasalazine, Mezalazine (oral forms) and enema and suppositories (topical forms); immunosuppressive drugs (18-20): Azathioprine and Prednisone; biological or anti-TNFα agents (21): Infliximab and Adalimumab. It should also be noted that Tofacitinib information was removed from the analysis in this study owing to the low number of prescriptions among patients.

***Statistical analysis***

First, the Markov property ”The system depended only on the current state in which it was located to change its state and not on its previous states” of our model was checked using the Markov chain package in R software version 3.6. Then, to delineate the IBD natural history, a continuous Markov time model in R's MSM package was applied, which allowed the patient's disease state to vary between states. To accomplish this, the model was fitted to the data before the parameters were estimated using the maximum likelihood function. Between states hazard ratios and transition probabilities were extracted from the fitted model (comprising sex, age, and drug family variables), and the mean sojourn time for each state except surgery (absorbing state) were estimated. 

A panel-ordered logistic regression was also performed to better explain the impact of covariate on disease severity (comprising remission, mild, moderate to severe, and surgery states). A random effect ordered logit developed in the PGLM package of the R program was used. Because IBD is a non-homogeneous disease with marked heterogeneity across patients (13), it was assumed that variations between and within individuals would be encountered. This assumption was tested using the variance component (Sigma), which revealed that the variability across units favored a random effect model. Throughout this paper, the criterion is clinical importance rather than statistical significance, because the sample size in this analysis is small, and most likely a sparse data bias exists, at least in the case of surgical transitions. In the case of chronic diseases such as IBD, however, the objective was to improve patients' quality of life and extend the period of remission. 

**Table 1:** Mean sojourn time (months) and CI: %95 for each state in UC patients

	Yes	No
Sex ^a^		
Remission	9.15(1.35-61.67)	11.87(1.99-70.61)
Mild	3.99(1.50-10.60)	3.73(1.55-8.98)
Moderate to severe	4.81(2.35-9.86)	6.87(3.43-13.75)
Anti TNFα agents		
Remission	12.05(2.13-62.80)	11.87(1.99-70.61)
Mild	2.69(1.06-6.85)	3.73(1.55-8.98)
Moderate to severe	4.26(1.83-9.87)	6.87(3.43-13.75)
5-ASA agents		
Remission	9.85(3.07-29.83)	11.87(1.99-70.61)
Mild	3.43(1.68-6.99)	3.73(1.55-8.98)
Moderate to severe	9.71(4.09-23.05)	6.87(3.43-13.75)
5-ASA agents(Topical)		
Remission	15.67(2.40-102.27)	11.87(1.99-70.61)
Mild	3.23(1.20-8.65)	3.73(1.55-8.98)
Moderate to severe	7.98(3.74-17.03)	6.87(3.43-13.75)
Immunosuppressive agents		
Remission	16.64(2.84-97.47)	11.87(1.99-70.61)
Mild	3.28(1.36-7.90)	3.73(1.55-8.98)
Moderate to severe	6.30(2.98-13.32)	6.87(3.43-13.75)
^a ^yes==female and no==male		

## Results

***Basic information and drug usage***

In this study, 134 UC patients and 64 CD patients participated. Among UC patients, 76 (56%) were male and 58 (44%) were female. Among CD patients, 29 (44%) were female and 36 (56%) were male. The maximum number of visits per patient in the clinic was 21 for ulcerative colitis and 16 for Crohn's disease. In CD patients, biological drugs were prescribed 265 times out of a total of 365 prescriptions; 5-ASA agents were prescribed 165 times, 5-ASA agents (topical) were prescribed 23 times and immunosuppressive agents 259 times. For UC patients, biological drugs were prescribed 247 times out of a total of 720 prescriptions; 5-ASA agents were prescribed 629 times, 5-ASA agents (topical) 193 times, and immunosuppressive agents 528 times.

***Multi-state and*** ***panel ordered logistic models***

Mean sojourn time, hazards ratio, and odds ratio were estimated to obtain IBD (UC/CD) natural history perspective. To correctly present the results of the previous estimates, an attempt was made to identify the results of these estimates for each of the factors examined together and separately for Crohn's disease and ulcerative colitis.

***Ulcerative colitis***

**Gender**

Longer sojourn time (about 2-3 months) for remission and moderate to severe states and a slightly shorter sojourn time for the mild state in males were observed (Table 1). Likewise, transitions to the mild state were slightly faster in women, especially from moderate to severe state (HR=1.490, 95%:1.02-2.16) (Table 3). The odds of the high level of disease severity were (OR=0.69, 95% CI: 0.40-1.19) lower for females than for males (Table 4). These findings demonstrate that women frequently had a mild form of the disease and a higher level of well-being.

**Table 2:** Mean sojourn time (month) and CI: %95 for each state of CD patients

	Yes	No
Sex ^a^		
Remission	14.31(0.68-301.07)	4.72(0.15-147.67)
Mild	8.49(1.07-67.18)	7.88(1.59-38.88)
Moderate to severe	9.98(3.54-28.13)	9.98(3.87-25.74)
Anti TNFα agents		
Remission	21.86(3.34-142.82)	14.31(0.68-301.07)
Mild	7.64(1.74-33.53)	8.49(1.07-67.18)
Moderate to severe	5.97(2.38-14.93)	9.98(3.54-28.13)
5-ASA agents		
Remission	17.89(0.73-436.29)	14.31(0.68-301.07)
Mild	6.52(0.56-74.75)	8.49(1.07-67.18)
Moderate to severe	9.89(3.07-31.89)	9.98(3.54-28.13)
5-ASA agents(Topical)		
Remission	0.78(0.01-38.21)	14.31(0.68-301.07)
Mild	5.77(0.71-46.60)	8.49(1.07-67.18)
Moderate to severe	15.98(3.94-64.73)	9.98(3.54-28.13)
Immunosuppressive agents		
Remission	10.66(0.55-203.03)	14.31(0.68-301.07)
Mild	11.15(2.21-56.26)	8.49(1.07-67.18)
Moderate to severe	7.95(2.93-21.52)	9.98(3.54-28.13)
^a ^yes==female and no==male		

**Table 3:** Health states hazard rations and CI:%95 for each covariates in IBD patients(UC/CD)

	Ulcerative colitis	Crohn's disease
Health states transition for covariates	Multivariate HR(95%,CI)	Multivariate HR(95%,CI)
Age		
Remission to Mild	1.001(0.97-1.02)	0.960(90-1.02)
Mild to Remission	0.979(0.95-1.02)	1.020(0.98-1.05)
Mild to Moderate to severe	0.981(0.95-1.01)	0.989(0.94-1.03)
Moderate to Severe to Mild	0.995(0.97-1.01)	0.991(0.97-1.01)
Moderate to Severe to Colectomy	1.062(1.09-1.12)	1.007(0.93-1.08)
Sex[male=0]		
Remission to Mild	1.297(0.70-2.38)	0.330(0.08-1.25)
Mild to Remission	1.195(0.74-1.91)	0.588(0.26-1.33)
Mild to Moderate to severe	0.788(0.43-1.42)	1.221(0.471-3.22)
Moderate to Severe to Mild	1.490(1.02-2.16)	1.002(0.54-1.85)
Moderate to Severe to Colectomy	0.022(0.00-57.54)	0.474(0.02-9.09)
Anti TNFα agents[No usage=0]		
Remission to Mild	0.985(0.53-1.81)	0.654(0.07-5.85)
Mild to Remission	1.365(0.86-2.16)	1.490(0.59-3.75)
Mild to Moderate to severe	1.395(0.79-2.44)	0.952(0.29-3.11)
Moderate to Severe to Mild	1.663(1.11-2.48)	1.588(0.82-3.05)
Moderate to Severe to Colectomy	0.502(0.04-5.61)	-
5-ASA agents (oral)[ No usage=0]		
Remission to Mild	1.239(0.28-5.37)	0.800(0.22-2.81)
Mild to Remission	1.780(0.68-4.65)	0.766(0.37-1.58)
Mild to Moderate to severe	0.699(0.31-1.57)	1.526(0.59-3.89)
Moderate to Severe to Mild	0.736(0.41-1.30)	1.008(0.56-1.80)
Moderate to Severe to Colectomy	0.078(0.009-0.66)	1.500(0.08-27.80)
5-ASA agents (topical)[ No usage=0]		
Remission to Mild	0.757(0.39-1.46)_	18.339(1.88-…)
Mild to Remission	0.882(0.52-1.47)	1.099(0.30-3.94)
Mild to Moderate to severe	1.310(0.72-2.38)	1.626(0.49-5.29)
Moderate to Severe to Mild	0.877(0.0.59-1.30)	0.625(0.21-1.81)
Moderate to Severe to Colectomy	0.509(0.05-4.42)	0.032(0.00-…)
Immunosuppressive agents [ No usage=0]		
Remission to Mild	0.713(0.38-1.33)	1.343(0.34-5.23)
Mild to Remission	1.258(0.75-2.09)	0.783(0.30-2.03)
Mild to Moderate to severe	1.070(0.59-1.93)	0.752(0.19-2.84)
Moderate to Severe to Mild	1.065(0.63-1.78)	0.752(0.61-2.58)
Moderate to Severe to Colectomy	1.631(0.20-13.16)	0.535(0.04-6.95)

**Table 4.** Results of random-effect ordered logistic regression for two IBD subtype (UC/CD)

Variables	UC	CD
Sex		
Male	1	1
Female	0.69(0.40-1.19)	1.26(0.57-2.77)
Age	1.00(0.97-1.02)	0.97(0.94-1.00)
Anti TNFα agents		
No	1	1
Yes	0.54(0.35-0.85)	0.10(0.04-0.23)
5-ASA agents (oral)		
No	1	1
Yes	0.19(0.09-0.40)	0.95(0.51-1.76)
5-ASA agents (topical)		
No	1	1
Yes	1.65(1.12-2.42)	0.87(0.29-2.85)
Immunosuppressive agents		
No	1	1
Yes	1.28(0.82-2.01)	1.00(0.52-1.94)

**Age**

Shorter transition rates from mild to remission (HR=0.979, 95%CI: 0.95-1.02), mild to moderate to severe (HR=0.981, 95% CI: 0.95-1.01), and moderate to severe to mild (HR=0.995, 95% CI: 0.97-1.01) were observed (Table 3). It appears that with increases in age, the rate of change between states decreased slightly.

**Biologic drugs**

Slightly longer sojourn time for remission, an almost one-month shorter time for mild, and about three months shorter sojourn time for moderate to severe states were observed in users of biologic drugs (Table 1). A moderately fast transition rate to states with a better quality of life, a faster rate from moderate to severe to mild (HR=1.663, 95% CI: 1.11-2.48), and a slower rate from moderate to severe to colectomy (HR=0.502, 95% CI: 0.04-5.61) were also observed (Table 3). 

Biologic users also had (OR=0.54, 95% CI: 0.35-0.85) lower odds of having a high level of disease severity (Table 4). These results confirm the prolonged remission time for biologic users.

**Oral 5-ASA drugs**

An approximately two-months shorter sojourn time was observed for oral 5-ASA users on remission and a longer time (about three months) for the moderate to severe state (Table 1). 

The results showed a faster transition from remission to mild (HR=1.239, 95% CI: 0.28-5.37) and from mild to remission (HR=1.780, 95%CI: 0.68-4.65) (Table 3) as well as (OR=0.19, 95% CI: 0.09-0.40) lower odds of having a high level of severity (Table 4). It seems that oral 5-ASA medications are most effective for people in remission and with mild conditions.

**Topical 5-ASA drugs**

A longer sojourn time was observed in remission (15.67 vs.11.87) and moderate to severe (7.98 vs. 6.87) states. Moreover, a faster transition rate to moderate to severe state was also observed (HR=1.310, 95% CI: 0.72-2.38) (Table 3). Higher odds (OR=1.65, 95% CI: 1.12-2.42) of having a high level of disease severity were observed in these patients (Table 4). These findings can support a prescription for non-concomitant use of 5-ASA drugs in UC patients who have escalated to biologic agents (22, 23).

**Immunosuppressive drugs**

An approximately five-month longer sojourn time for remission and slightly better sojourn time for mild (3.28 vs. 3.73) and moderate to severe (6.30 vs. 6.87) states were observed. Also observed were a slower transition rate from remission to mild (HR=0.713, 95% CI: 0.38-1.33), a faster transition from mild to remission (HR=1.258, 95% CI: 0.75-2.09), and a faster transition for colectomy. Higher odds (OR=1.28, 95% CI: 0.82-2.01) of having a high level of disease severity in these patients was also observed (Table 4). These results can support multiple therapies and not monotherapy only with immunosuppressive drugs, especially for patients in moderate to severe states.

***Crohn’s disease***

**Gender**

Longer sojourn times for remission (14.31 vs. 4.72) and mild (8.49 vs. 7.88) states and equal time to stay in the moderate to severe state at 9.98 were observed in women (Table 2). Women were also found to have a slower shift from remission to mild (HR=0.330, 95% CI: 0.08-1.25) and a slightly faster transition from mild to moderate to severe states (HR=1.221, 95% CI: 0.471-3.22) (Table 3) as well as higher odds (OR=1.26, 95% CI: 0.57-2.77) of having a high level of dependent variables compared to men (Table 4). Based on these results, it can be claimed that women experience a prolonged remission.

**Age**

With increases in age, the remission period increased slightly (remission to mild: HR=0.960, 95% CI: 90-1.02 vs. mild to remission: HR=1.020, 95% CI: 0.98-1.05) (Table 3).

**Biologic drugs**

A longer sojourn time for remission (21.86 vs. 14.31) and a shorter sojourn time for mild (7.64 vs. 8.49) and moderate to severe states (5.97 vs. 9.98) were observed (Table 2). Moreover, a slower transition from remission to mild state (HR=0.654, 95% CI: 0.07-5.85) and a faster transition from mild to remission state (HR=1.490, 95% CI: 0.59-3.75) were seen (Table 3). They also experienced the same former pattern between mild and moderate to severe states (Table 3). These patients had (OR=0.10, 95% CI: 0.04-0.23) lower odds of having a high level of disease severity. These findings demonstrate longer remission times in biologic users. 

**Oral 5-ASA drugs**

In terms of mean sojourn time, a very similar trend to that of biological users was observed, especially for remission (Table 2). Moreover, relatively the same transition rate was observed between remission and mild states and faster transition from mild to moderate to severe and from moderate to severe state to surgery (HR=1.500, 95% CI: 0.08-27.80) (Table 3). Relatively lower odds (OR=0.95, 95% CI: 0.51-1.76) of having a high level of disease severity were also observed (Table 4). These results suggest that oral 5-ASA is preferable for patients in remission and mild states but not for those in moderate to severe state.

**Topical** **5-ASA drugs**

A shorter sojourn time was observed for remission (0.78 vs. 14.31) and mild (5.77 vs. 8.49) states, and a longer sojourn time was seen for moderate to severe state (15.98 vs. 9.98) (Table 2). Moreover, a faster transition was observed for states with poor prognosis in this patient (Table 3). Relatively lower odds (OR=0.87, 95% CI: 0.29-2.85) of having a high level of disease severity were also seen (Table 4). In general, the current results showed that topical 5-ASA drugs are ineffective for stretching the remission phase in CD patients.

**Immunosuppressive drugs**

In these patients, remission (10.66 vs. 14.31) and moderate to severe (7.95 vs. 9.98) states were observed to have shorter sojourn times, while mild state had longer sojourn times (11.15 vs. 8.49) (Table 2). A faster transition from remission to mild (HR=1.343, 95% CI: 0.34-5.23) than from mild to remission was also observed (Table 4). Immunosuppressive users had an equal mutual transition between mild and moderate to severe states and a slower transition rate to colectomy. These users had (OR=1.00, 95% CI: 0.52-1.94) odds of having a high level of severity compared to non-users of immunosuppressive drugs. The current findings suggest that combining these medications with other drug groups provides a better outcome than monotherapy (especially for moderate to severe state).

## Discussion

In general, the current results confirm that panel approaches potentially have efficacy in tackling the unpredictable clinical course of IBD (UC/CD) by providing a better understanding of treatments and the effects of covariates and their subsequent outcomes. The prominent disease status in women was mild for UC and they also experienced prolonged remission for CD. Biologic drugs delayed the transition to a state with poor quality of life (non-prolonged remission time). Oral 5-ASA drugs were most effective for patients in remission and mild state but not for those in a moderate to severe state in both UC and CD patients. The findings provided evidence for a recommendation of the use of non-concomitant topical 5-ASA drugs in patients who have escalated to using biologic agents owing to a quicker transition and higher odds of a poorer quality of life in UC patients. Topical 5-ASA was also found not to help extend the period of remission in CD patients. Immunosuppressive drugs have better efficacy with multiple therapy (not monotherapy), particularly in moderate to severe IBD patients (UC/CD). Overall, it is recommended that gastroenterologists consider the current findings in clinical environments to enhance disease management. 

For UC, men experienced higher transition and/or sojourn time for remission state, which is congruent with other evidence (24, 25), but women had a slightly better condition for moderate to severe state. In CD patients, prolonged remission of women and poor condition for moderate to severe state are again concordant with other studies (24, 25) that have stated that active patients (not in remission) were often female. Moreover, when compared to the findings of random effect ordered logistic, MSM performs better, because it more accurately shows the condition of women in both remission and severe stages and distinguishes between them. The data demonstrates that the severity of IBD (UC/CD) differs between men and women, which should be known in order to enhance clinical management.

As expected, in the biologic era (26, 27), an increase in the quality of life (prolonged remission) of IBD (UC/CD) patients treated with biologic agents was observed, particularly in CD patients. However, evidence revealed that IBD patients, even in the current situation, are still facing surgery in their lifetime (26, 28). Moreover, these drugs are ineffective for a large proportion of patients (27). However, the current results suggest using these drugs for the management of IBD.

There is adequate evidence (29, 30) to support the more effectiveness of oral 5-ASA drugs (as frontline therapy of IBD) for people with remission and/or mild states but not for moderate to severe states, especially in CD. Recommendations discouraging the use of 5-ASA for active Crohn's disease is available in the literature (31). In this regard, a faster transition to surgery for CD patients but slower for UC was observed. However, the evidence is somewhat conflicting and supports the greater effect of oral 5-ASA in UC than in CD (32, 33).

The current results provide evidence to confirm the recommendation for non-concomitant 5-ASA drugs in UC patients who have escalated to using biologic agents (22, 23), which must be considered by clinicians. Studies have also shown that combining oral and rectal ASA drugs is more effective for UC patients (34), which confirms the results of the current study. The recommendation not to use them in Crohn's disease in either rectal or ileal form, as demonstrated by the literature, was also confirmed by the current study (34). Faster transition rates, higher odds, and longer sojourn times were observed for states with poor quality of life (non-prolonged remission time). Therefore, it seems that they should be used very carefully in the treatment of Crohn's disease.

The current results also provide evidence that combining immunosuppressive agents with other drugs (multiple therapies) result in better outcomes than monotherapy in IBD patients (UC/CD) (especially for moderate to severe state). One clinical trial research supports these findings (35). Thus, higher remission and mucosal healing rates were found in CD patients in the combination arm. For UC, the supportive evidence usually emphasizes remission rather than mucosal healing (36).

The limitations of the current study include the fact that, due to lack of access, the effects of some significant modifiers, such as smoking, stress, depression, and anxiety, have been overlooked. On the other hand, while the sample size in the current study was small, to the best of the authors’ knowledge, this study is the first attempt to explain IBD's natural history by applying panel approaches, considering common medicines in Iranian routine clinical settings.

To get a general overview and check the external validity of the current results, they were compared with the American Gastroenterological Association (AGA) Clinical Guidelines for IBD (UC/CD) (37, 38). AGA recommendations were consistent with the current results regarding the use of oral 5-ASA drugs for people with remission or/and mild states but not for moderate to severe state (conditional recommendation, low level of evidence), multiple therapies with immunosuppressive drugs, especially for moderate to severe state (strong recommendation, high level of evidence), and use of biologic drugs for the management of IBD (strong recommendation, moderate level of evidence).

Taken together, the current findings can be used in the routine IBD clinical environment in Iran, and this is strongly suggested. It is also strongly advised that similar research be conducted with a deeper and more critical perspective on diagnostic procedures, disease modification, and the advantages and problems of novel and traditional medical therapies so as to obtain suitable clinical advice for treating IBD in Iran. To the best of the authors’ knowledge, this is the first study to investigate the natural history of IBD. Hence, doing similar research in other parts of the world can be incredibly beneficial.

## Conflict of interests

The authors declare that they have no conflict of interest.
